# MITOGENOME ANNOUNCEMENTSCharacterization of the complete chloroplast genome of the Chinese endangered species *Cymbidium iridioides* D. Don

**DOI:** 10.1080/23802359.2021.1964398

**Published:** 2021-08-17

**Authors:** Longjie Cheng, Yantao Xu, Zhilin Li, Yiran Zhao, Fengmei He, Yuying Wang

**Affiliations:** a College of Horticulture and Landscape, Yunnan Agricultural University, Kunming, China; b Forestry Bureau of Jimo City, Jimo, China

**Keywords:** *Cymbidium iridioides*, chloroplast genome, endangered species, phylogenetic analysis

## Abstract

*Cymbidium iridioides* D. Don 1852 is a Class I endangered species in China having important ornamental and breeding values. This study used Illumina high-throughput sequencing technology to sequence the complete chloroplast genome of *C. iridioides.* The genome features of *C. iridioides* and its phylogenetic relationships were determined. The complete chloroplast genome is 156,599 bp, containing a pair of 26,736 bp inverse duplication regions, a large 85,242 bp single-copy region, and a small 17,885 bp single-copy region. The entire genome contains 76 protein-coding genes, 37 tRNA genes, and 4 rRNA genes. A phylogenetic tree of 25 Orchidaceae species revealed that *C. iridioides* was grouped within Sect. Iridorchis and comprised a clade with *Cymbidium tracyanum*.

There are approximately 60 species of *Cymbidium* in the world (Liu et al. 2006). *Cymbidium iridioides* D. Don 1852 (Orchidaceae) is a herb that is distributed in SW Guizhou, SW Sichuan, SE Xizang, NW to SE Yunnan of China. It is epiphytic on trunks or branches at altitudes of 900–2800 m. *Cymbidium iridioides* has been listed as a Class I protected plant in the China Rare and Endangered Plants List (http://www.iplant.cn/rep/protlist). It has 3–17 fragrant flowers, yellowish green sepals and petals with 7–9 brownish or reddish brown longitudinal stripes and yellowish lip with reddish brown stripes on lateral lobes and similarly colored mottling on midlobe. The flowering period is long, ranging from August to December (Liu et al. 2009). Owing to its beautiful flowers and long inflorescence, *C. iridioides* is a vitally important germplasm resource for potted flowers, cut flowers, and flower breeding.

The complete chloroplast genome sequence of *C. iridioides* was obtained (GenBank Accession No. MZ044639). The genome sequence and features aid in determining the phylogenetic relationships of *C. iridioides* and for the in-depth study of chloroplasts. In addition, this genome is important for studying the diversity of the plant’s genetic sources. The leaf sample of *C. iridioides* was gathered from the Flower Research Institute of the College of Horticulture and Landscape, Yunnan Agricultural University, Kunming, Yunnan Province, China (25°07′43″ N, 102°44′54″ E). The voucher specimens and DNA were deposited at Herbarium of Kunming Institute of Botany of CAS (Yuying Wang, wyysxp@126.com) under the voucher number CY006. The entire chloroplast DNA was extracted with the CTAB (Cetyltrimethyl Ammonium Bromide) protocol (Doyle and Doyle 1987) from fresh mesophyll tissue of *C. iridioides.*

DNA sequencing was performed using Illumina NovaSeq at GENOSEQ Technologies Limited (Wuhan, China). The raw data were obtained and then assembled using SPAdes (version: 3.13.0) (Bankevich et al. 2012). The assembled contigs were compared with the chloroplast genomes of the closely related species by using BLASTN (version: BLAST 2.2.30+; parameter: -evalue 1e-5). Then, the contigs were checked, selected and adjusted to acquire the final data. The chloroplast genome was annotated, and mapped using CPGAVAS2 (Linchun et al. 2019). The raw sequencing reads were deposited in the SRA (PRJNA725276).

The length of the complete chloroplast genome of *C. iridioides* is 156,599 bp. The genome presented a characteristic quadripartite circular structure, which included one pair of inverted repeat regions (IRs, 26,736 bp), one large single-copy region (LSC, 85,242 bp), and one small single-copy region (SSC, 17,885 bp). In addition, the complete genome contains 76 protein-coding genes, 37 transfer RNA genes, and 4 ribosomal RNA genes. The overall GC content of the *C. iridioides* chloroplast genome is 36.77%. Moreover, with the GC content of the IR regions (43.11%) being higher than those of the LSC (34.28%) and SSC (29.72%) regions. rRNAs only exist in the IR region. In total, 18 genes are repeated in the IR region, 6 protein-coding, 8 tRNA, and 4 rRNA. The ycf1 gene is at the junction between the IRA and SSC. The matK geneis is located in trnK-UUU.

To study the phylogenetic relationships of *C. iridioides*, a phylogenetic tree was constructed using 22 complete chloroplast genomes of *Cymbidium* species, and three *Orchidaceae* species were selected as outgroups. All the sequences were downloaded from NCBI GenBank. All the sequences were aligned using the online program MAFFT version 7.0, and MEGA version 7.0 was used to build the maximum-likelihood phylogenetic tree with 1000 rapid bootstrap replicates (Kumar et al. 2016). The phylogenetic tree analysis indicated that *C. iridioides* was grouped within Sect. Iridorchis and comprised a clade with *Cymbidium tracyanum* (Figure 1).

**Figure 1.**
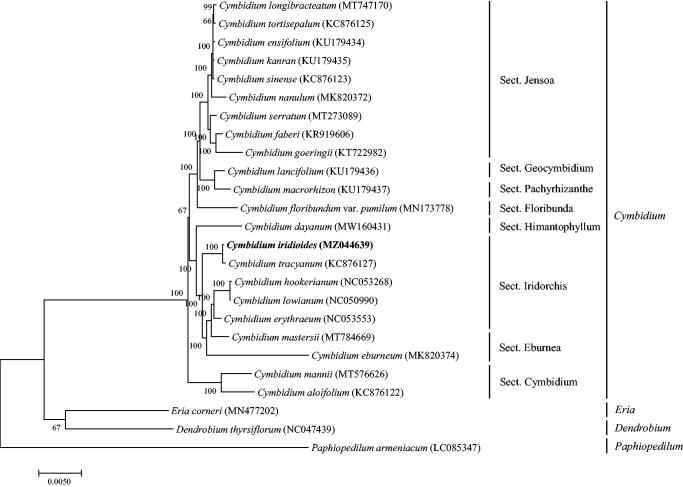
A Phylogenetic tree based on 25 complete chloroplast genome sequences of Orchidaceae species using the Maximum Likelihood (ML) analysis by MEGA version 7.0. Bootstrap support values are indicated in each node.

## Data Availability

The data that support the findings of this study are openly available in GenBank of NCBI at https://www.ncbi.nlm.nih.gov, reference number MZ044639. The associated BioProject, SRA and Bio-Sample numbers are PRJNA725276, SRP316447 and SAMN18875809 respectively.
